# Finding microRNA regulatory modules in human genome using rule induction

**DOI:** 10.1186/1471-2105-9-S12-S5

**Published:** 2008-12-12

**Authors:** Dang Hung Tran, Kenji Satou, Tu Bao Ho

**Affiliations:** 1 School of Knowledge Science, Japan Advanced Institute of Science and Technology, 1-1 Asahidai, Nomi, Ishikawa 923-1292, Japan; 2 Kanazawa University, Kakuma, Kanazawa 920-1192, Japan

## Abstract

**Background:**

MicroRNAs (miRNAs) are a class of small non-coding RNA molecules (20–24 nt), which are believed to participate in repression of gene expression. They play important roles in several biological processes (e.g. cell death and cell growth). Both experimental and computational approaches have been used to determine the function of miRNAs in cellular processes. Most efforts have concentrated on identification of miRNAs and their target genes. However, understanding the regulatory mechanism of miRNAs in the gene regulatory network is also essential to the discovery of functions of miRNAs in complex cellular systems. To understand the regulatory mechanism of miRNAs in complex cellular systems, we need to identify the functional modules involved in complex interactions between miRNAs and their target genes.

**Results:**

We propose a rule-based learning method to identify groups of miRNAs and target genes that are believed to participate cooperatively in the post-transcriptional gene regulation, so-called miRNA regulatory modules (MRMs). Applying our method to human genes and miRNAs, we found 79 MRMs. The MRMs are produced from multiple information sources, including miRNA-target binding information, gene expression and miRNA expression profiles. Analysis of two first MRMs shows that these MRMs consist of highly-related miRNAs and their target genes with respect to biological processes.

**Conclusion:**

The MRMs found by our method have high correlation in expression patterns of miRNAs as well as mRNAs. The mRNAs included in the same module shared similar biological functions, indicating the ability of our method to detect functionality-related genes. Moreover, review of the literature reveals that miRNAs in a module are involved in several types of human cancer.

## Background

MicroRNAs (miRNAs) are a class of small non-coding RNA molecules (20-24 nt), which are believed to participate in down-regulation of gene expressions. They inhibit their target genes (mRNA) in the post-transcriptional process by complementary base pairing [1-3]. Currently, 475 human miRNAs have been annotated in the miRNA registry, with over 1,000 miRNAs predicted to exist in humans. These miRNAs are predicted to target one-third of all genes in the genome, where each miRNA is expected to target around 200 transcripts [4,5]. Recent studies have shown that miRNA can play fundamentally important roles in animal and plant development [1-3] as well as in genetic diseases including various types of cancer [6-9]. Therefore, discovering the functions of miRNA in living cells is an important task in biology.

Up to now, researchers have made many attempts to understand the functions of miRNAs in cellular processes more clearly, using both experimental and computational methods. Most efforts have concentrated on finding miRNAs and their targets [10-13]. However, understanding the regulatory mechanism of miRNAs in the gene regulatory network is also essential to the discovery of functions of miRNAs in complex cellular systems. In animal cells, miRNA regulatory mechanism is represented by the relationships between miRNAs and their targets at the post-transcriptional level of the gene regulation network. Furthermore, the relationship between miRNAs and their target genes is generally complicated. One target gene could be regulated by several miRNAs and conversely, one miRNA may have several target genes [1,2,7].

In order to understand the regulatory mechanism of miRNAs in complex cellular systems and to discover important patterns hidden in the complex interactions, we need to identify the functional modules involved in complex interactions between miRNAs and their target genes [14,15]. Previously, Yoon and De Micheli introduced the concept of miRNA regulatory modules (MRMs) [15], which are defined as groups of miRNAs and their target genes that are believed to have similar functions or to be involved in similar biological processes. They represented the multiple relations between miRNAs and target genes by a weighted bipartite graph, and then used a five-step method to find MRMs [15]. The main drawback of their method is that it deals only with miRNA-mRNA duplexes at the sequence level. Using only this kind of information may not be sufficient for determining MRMs. Other information such as miRNA and mRNA expression profiles could be also useful to detect the natural MRMs in a specific biological process [16,17]. Another approach, proposed by Joung *et al. *[14], tries to combine multiple information sources to extract the MRMs. This method, however, relies on a genetic algorithm that undergoes several random processes. Therefore, the quality of their result depends on many sensitive parameters, thus making it unreliable.

As we know that miRNAs regulate expression by binding to cis-regulatory regions of 3'-UTR regions of genes, it is therefore reasonable to assume that genes regulated by the same miRNAs should contain similar expression profiles. This assumption initializes our analysis of human miRNA-target binding data and gene expression data to reveal the combinatorial nature of gene regulation at the post-transcription level. In this paper, we present a new computational method using rule learning to perform a comprehensive analysis of the combinatorial nature of gene regulation by detecting rules that identify a set of miRNAs associated with genes. The method extracts IF-THEN rules of miRNA combinations shared by target genes with a common expression profile. Similar to the approach of Joung *et al. *[14], our method also uses multiple information sources, including miRNA-target binding information, gene expression and miRNA expression profiles. However, the rule learning method allowed us to find the combinatorial nature of miRNA regulatory network without using any random process. As a result, the MRMs, found by our method, consist of highly-related miRNAs and their target genes with respect to biological processes. Moreover, evaluating MRMs by using the literature suggests that miRNAs in a module are involved in several types of cancer, and genes in the module indeed share common roles in biological processes.

## Results and discussion

### Finding potential miRNA regulatory modules

We applied our method to the human miRNA dataset as described in the Section *Datasets*. Table 1 shows the summary of potential MRMs induced by our method after applying several filtering procedures (Section *Filtering rules*). In general, the rule induction system can produce many rules from the miRNA regulatory table for each target gene. It may be that not all of them are interesting (i.e. significant with respect to biological processes). For finding the rules regarding highly related miRNAs and target genes with respect to expression, we used the Pearson's coefficient correlation (PCC) to remove uninteresting rules. A rule is significant if the PCC between any two genes is greater than a threshold (column 1, Table 1), and the same threshold was applied to miRNAs in that rule as well.

**Table 1:** Summary of miRNA regulatory rules induced by our method (confidence ≥ 0.75 and coverage ≥ 3)

PCC^*a*^	#Rule	#miRNA^*b*^	#miRNA_target^*c*^
0.1	81	2.4	5.2
0.2	79	2.4	4.6
0.3	54	2.2	4.2
0.4	36	2.2	3.6
0.5	27	2.0	3.1

^*a *^The Pearson's coefficient correlation.

^*b *^The number of miRNAs on average in each regulatory rule.

^*c *^The number of target genes on average in each regulatory rule.

We evaluated rules using the concept of *confidence *and *coverage*. *Confidence *indicates the exactness of the rules and defined as *confidence *= *p*/*P*; where *p *is the number of examples of positive class (i.e. *similarity *class) covered by the rule, and *P *is the number of all examples in the dataset covered by the rule. *Coverage *indicates the generality of the rules (i.e. the number of examples of positive class covered by the rule) and defined as *coverage *= *p*. Rule induction may produce a large number of very specific rules (i.e. rules with low *coverage*), indicating that no general relationship could be found between miRNA-binding information and expression data for these target genes. Other rules will cover many genes with a large diversity in their expression profiles (i.e. rules with low accuracy), violating the assumption that genes regulated by the same miRNAs should be coexpressed. Only when we find miRNA combinations common to several target genes with similar expression may we expect a high probability for actual coregulation.

In order to get a good estimate of our ability to find biologically interesting MRMs, we induced rules using only the 121 known miRNAs in human (Section *Datasets*). The number of rules induced from the dataset is given in column 2 in Table 1. The fact that our rule learning algorithm finds minimal miRNA combinations is attractive in general (column 3, Table 1). It also can be seen that our method produced fewer rules, when compared to previous methods (see [14] and [15]). The reason is that expression patterns of miRNAs as well as mRNAs in our rules were highly correlated. From each miRNA regulatory rule, we can easily obtain one corresponding potential MRM by finding *similarity *class examples covered by this rule. Table 2 shows thirty selected MRMs were found when our method was applied to the dataset mentioned in the Section *Datasets*. Due to limitations of space we can not show all modules, and the full set of potential MRMs can be obtained from our supplementary file .

**Table 2:** Examples of potential miRNA regulatory rules (PCC = 0.2)

Rule#	miRNAs	Target_genes	Confidence	Coverage
1	[hsa-miR-143, hsa-miR-181a]	[NOVA1, ST8SIA4, ZFP36L1]	1.00	3
2	[hsa-miR-125b, hsa-miR-145]	[DAG1, NEDD9, YES1, BMPR2, PTPRF]	0.86	5
3	[hsa-miR-126, hsa-miR-181b]	[PCAF, NOVA1, EIF4A2]	0.75	3
4	[hsa-miR-155, hsa-miR-27b]	[NOVA1, ZNF238, WEE1, ELL2, MAP3K14, PKIA, APC, ADD3]	0.86	8
5	[hsa-miR-27a, hsa-miR-143,	[NOVA1, CDH5, ADD3]	1.00	3
6	[hsa-miR-101, hsa-miR-19a, hsa-miR-221]	[ATXN1, CTCF, RAB1A]	1.00	3
7	[hsa-let-7e, hsa-miR-26a]	[ARID3A, TAF5, HAS2, NOVA1, AKAP6, DYRK1A]	0.86	6
8	[hsa-miR-149, hsa-miR-29a]	[BCL2L2, PLAG1, SP1, CBX1]	1.00	4
9	[hsa-miR-17-5p, hsa-miR-25]	[CIC, EDG1, SSFA2, PCAF, SALL1]	0.92	5
10	[hsa-miR-134, hsa-miR-15a]	[KPNA3, RUNX1T1, EPHA7]	0.75	3
12	[hsa-miR-15a, hsa-miR-216]	[DYRK1A, MAPRE1, BCL9]	1.00	3
13	[hsa-miR-199b, hsa-miR-26a]	[ZNF238, EPHA7, CDH2]	1.00	3
14	[hsa-let-7d, hsa-miR-125a]	[PRDM2, DOCK3, DPF2]	0.85	3
15	[hsa-miR-155, hsa-miR-30d]	[SOCS1, NOVA1, NR2F2, PAPOLA, ELL2]	0.96	5
16	[hsa-miR-182, hsa-miR-205]	[DYRK1A, MMD, YES1, MAPK9, SMAD1]	1.00	5
17	[hsa-miR-222, hsa-miR-29a]	[PLEKHC1, PTEN, INA]	0.87	3
18	[hsa-miR-182, hsa-miR-183]	[YES1, SLC35A1, FGF9]	0.75	3
19	[hsa-miR-205, hsa-miR-30d]	[MMD, CAPZA1, SMAD1]	0.90	3
20	[hsa-miR-142-3p, hsa-miR-200c]	[MMD, PCAF, ANK3, ADAMTS3]	1.00	4
21	[hsa-miR-17-5p, hsa-miR-205]	[DYRK1A, YES1, BAMBI, MKNK1]	0.8	4
22	[hsa-miR-106b, hsa-miR-146]	[EGR3, RARB, MAP3K8]	1.00	3
23	[hsa-miR-103, hsa-miR-182]	[BCL2L2, MAP7, SRPK1, SMAD7]	0.79	4
24	[hsa-miR-142-5p, hsa-miR-27a]	[CACNB2, CLCN3, UBE4A, PPM1G]	1.00	4
25	[hsa-miR-101, hsa-miR-218, hsa-miR-22]	[FBN2, TLK2, BCL9]	0.82	3
26	[hsa-miR-181c, hsa-miR-18]	[ATP2B1, ATXN1, PLAG1, ESR1]	1.00	4
27	[hsa-miR-133a, hsa-miR-153]	[RANBP2, GNAI3, POU4F1, CDC2L5]	1.00	4
28	[hsa-miR-137, hsa-miR-142-5p]	[NR3C2, ATP1B1, CUL4A]	1.00	3
29	[hsa-miR-122a, hsa-miR-30e]	[MAPRE1, MAP3K12, PAPOLA]	0.79	3
30	[hsa-miR-138, hsa-miR-183]	[EPHA4, TRAM1, RCN2]	1.00	3

miRNA regulatory modules were selected from the full list of 79 modules, to be shown as examples. The Pearson's correlation coefficient between any gene pairs (as well as any miRNA pairs) in the same module was 0.2 or more.

**Figure 1:**
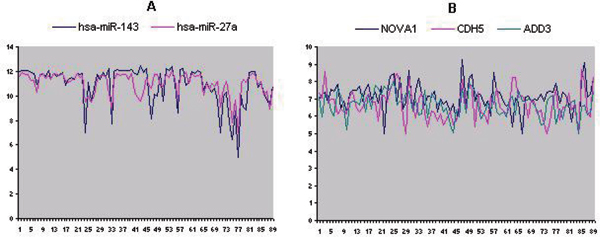
**Expression profiles of a module consists of two miRNAs and three target genes**. (A) Expression profiles of miRNAs; (B) Expression profiles of target genes. X-axis represents samples; Y-axis represents expression values. The expression data was obtained from [9] on 89 samples.

We also analyzed the expression patterns of miRNAs and mRNAs in each MRM, for example, Figure 1 shows the expression profiles of miRNAs and mRNAs of one MRM module that contains two miRNAs (hsa-miR-143 and hsa-miR-27a) and three target genes (NOVA1, CDH5, and ADD3). We can see that the expression patterns of miRNAs (Figure 1A) and mRNAs (Figure 1B) are highly similar. The illustration of expression patterns of other modules is omitted due to space limitations but can be performed in a similar manner.

### Validation using gene ontology

With the current knowledge of combinatorial coregulation, it is hard for us to directly validate potential MRMs. Fortunately, using Gene Ontology (GO) [18] we can validate the target genes in each MRM with respect to biological processes, cellular components and molecular functions. This validation can be achieved by searching for statistically significant GO terms.

In order to test if the target genes for each MRM might be enriched functionally based on arbitrary GO terms, we performed GO annotation and significance analysis using GOstat [19]. We observed terms associated significantly with the target genes included in the GO gene-association database (goa_human and Affymetrix HG_U95AV2 Human known genes). We also used the default setting of GOstat. To find significantly overrepresented GO terms, GOstat calculates a *P*-value upon assuming hyper-geometric distribution of annotated GO terms. Table 3 shows the significant *P*-values of the genes in our example modules. It can be seen that miRNA target genes in our modules are actually highly correlated on GO annotations.

**Table 3:** Biological processes of potential miRNA regulatory modules annotated in GO [18]

Module	GOid	Biological processes	Target genes	*P*-value
1	GO:0032501	Multicellular organismal process	NOVA1, ST8SIA4, ZFP36L1	8.63E-03
	GO:0009059	Macromolecule biosynthetic process	ST8SIA4, ZFP36L1	8.19E-03
2	GO:0007166	Cell surface receptor linked signal transduction	NEDD9, BMPR2, PTPRF	7.16E-03
	GO:0019538	Protein metabolic process	DAG1, YES1, BMPR2, PTPRF	7.16E-03
	GO:0006464	Protein modification process	YES1, BMPR2, PTPRF	7.16E-03
3	GO:0010467	Gene expression	PCAF, NOVA1, EIF4A2	7.49E-03
	GO:0018076	N-terminal peptidyl-lysine acetylation	PCAF, EIF4A2	5.65E-03
4	GO:0051348	Negative regulation of transferase activity	APC, PKIA	2.48E-03
	GO:0006469	Negative regulation of protein kinase activity	APC, PKIA	2.48E-03

Biological processes of four example modules were found by GOstat program [19]. GOid is the identification of the Gene Ontology (GO) term. *P*-values were calculated upon assuming hyper-geometric distribution of annotated GO terms.

### Supporting evidence of miRNA associated with cancers

Recent studies have shown that several miRNAs are directly involved in human cancers (including lung, breast, brain, liver, and colon cancer) [20-22]. This is because more than 50% of miRNA genes are located in cancer-associated genomic regions or fragile sites [23]. This evidence suggests that miRNAs may play a more important role in human cancers than was previously thought. Therefore, we validated the found modules with supporting evidence from the literature. Interestingly, several modules have been confirmed to be related to lung and other human cancers. For example, module 1 contains two miRNAs (hsa-miR-143 and hsa-miR-181b) and three target genes (NOVA1, ST8SIA4, and ZFP36L1). Both hsa-miR-143 and hsa-miR-181b are related to colorectal cancer [24,25]. Specifically, Micheal *et al. *[24] reported that hsa-miR-143 had decreased expression in both tumorigenic and precancerous tissues compared to normal samples. Several cancer cell lines (including colorectal adenocarcinoma and breast carcinoma) were also found to have decreased expression levels of hsa-miR-143 [24]. The expression level of hsa-miR-181b was investigated in the study of Xi *et al. *[25]. Their analysis revealed that hsa-miR-181b had high expression level in tumors displaying *p*53 deletion, and hsa-miR-181b expression level was strongly associated with the mutation status of the *p*53 in tumor.

Of these target genes in this module, NOVA1 encodes a neuron-specific RNA-binding protein, a member of the Nova family of paraneoplastic disease antigenes that is recognized and inhibited by paraneoplastic antibodies. These antibodies are found in the sera of patients with breast cancer and small cell lung cancer [26]. ST8SIA4 encodes a type II membrane protein, which is a member of glycosyltransferase family 29 and may be present in the Golgi apparatus. Although this gene is considered as a member of genes coding for membrane protein, it can show differences in expression levels between malignant and non-malignant tumor [27]. The last one, ZFP36L1, is a member of the TIS11 family of early response genes. This gene is well conserved across species and has a promoter that contains motifs seen in other early-response genes. It may have a role as an oncogene.

Module 2 consists of two miRNAs (hsa-miR-145 and hsa-miR-125b) and five target genes (DAG1, NEDD9, YES1, BMPR2, and PTPRF). Iorio *et al. *[28] analyzed the expression of 76 breast cancer and 10 normal breast samples to identify miRNAs whose expressions are significantly deregulated in cancer versus normal breast tissues. They reported that hsa-miR-125b and hsa-miR-145 were indeed involved in human breast cancer [28]. While hsa-miR-125b was down-regulated, hsa-miR-145 was up-regulated in human breast cancer. Their analysis suggested that these miRNAs may potentially act as tumor suppressors. Furthermore, expression of hsa-miR-145 was found at a low level in lung cancer tissues compared to normal samples [29]. Based on the target prediction and expression level of hsa-miR-145 in human cancers, Akao *et al. *[30] also suggested that this miRNA may suppress genes involved in signal transduction and oncogenesis.

Of five target genes in this module, three of them (NEDD9, BMPR2, and PTPRF) are involved in cell surface receptor linked signal transduction, and others are involved in protein metabolic process in terms of GO categories (Table 3). Interestingly, all genes also have roles in development of several type of cancers. For example, PTPRF encoded proteins which are known to be signaling molecules that regulate a variety of cellular processes including cell growth, differentiation, mitotic cycle, and oncogenic transformation. The PTPRF gene also plays important roles in colorectal cancers [31] and kidney carcinomas [32]. Therefore, it is reasonable for us to conclude that our predicted MRM modules are really related to human cancers.

Additionally, Table 4 shows several selected miRNAs from the set of our MRMs associated with human cancers. Based on overall investigation into recently published papers in the literature, we found that some miRNAs in our modules were confirmed as tumor suppressors while some other had function as oncogenes. This suggests that our method could be used to find potential miRNAs which may be associated with human cancers.

**Table 4:** Selected miRNAs associated with human cancers

miRNA	Function	Type of cancer	References
hsa-miR-143	Tumor suppressor	Colorectal, colon and breast cancer	[24]
hsa-miR-27b	Tumor suppressor	Colon cancer	[25]
hsa-miR-145	Tumor suppressor	Breast cancer	[24,28]
hsa-miR-125b	Tumor suppressor	Breast cancer, Hodgkin lymphoma	[28,40]
hsa-miR-155	Oncogene	Breast colon, and lung cancer	[28,41]
hsa-miR-17-5p	Oncogene	MYC, Lung cancer and B-cell lymphomas	[42]
hsa-miR-15a	Tumor suppressor	B-cell chronic lymphocytic leukemia	[43]
hsa-miR-221	Tumor suppressor	Papillary thyroid carcinoma, lung cancer	[40,42,44]
hsa-miR-181b	Tumor suppressor	Colorectal and colon cancer	[25,45,46]
hsa-miR-19a	Tumor suppressor	B-cell lymphoma	[42]
hsa-miR-200c	Tumor suppressor	Papillary thyroid carcinoma, B-cell lymphoma, colorectal cancer	[42,46]
hsa-miR-222	Oncogene	Papillary thyroid carcinoma	[42]
hsa-miR-146	Oncogene	Papillary thyroid carcinoma, breast cancer	[41,42]
hsa-miR-26a	Tumor suppressor	Colorectal cancer	[46]
hsa-miR-25	Tumor suppressor	Conlon cancer	[25,41]
hsa-miR-181a	Unknown	Acute myeloid leukaemia	[47]
hsa-miR-126	Tumor suppressor	Breast cancer metastasis	[48]
hsa-let-7d/e	Tumor suppressor	Lung cancer	[49]
hsa-miR-27a	Oncogene	Breast cancer	[50]
hsa-miR-125a	Tumor suppressor	Breast cancer	[28]

Several miRNAs in our module set were confirmed to be related to human cancers (including breast, lung, colon, and colorectal cancer).

## Conclusion

Although numerous miRNAs have recently been discovered in some species, their precise functional roles in cellular processes are still largely unknown. Specifically, the relationships between miRNAs and their target genes are less understood. In this paper we introduced a new computational method for finding MRMs from their predicted target genes and expression datasets (mRNA expression profiles and miRNA expression profiles). By combining these information sources, we can discover relevant MRMs in human genome.

In MRMs, found by our method, expression patterns of miRNAs as well as mRNAs were highly correlated. The mRNAs included in the same module also shared similar biological functions, indicating the ability of our method to detect functionality-related genes. Moreover, we also analyzed the relationships between several cancer diseases and our MRMs by using the literature. This analysis revealed that miRNAs in a module are involved in several types of cancer and genes in the module indeed share common roles in biological processes.

Despite these benefits of our method, several issues require further investigation. First, our rule induction method still produces a lot of rules. Many of them may be insignificant. New rule evaluation heuristic approaches could be used to reduce the search rule space. Second, the quality of MRMs obtained by our method depends on the choice of the similarity measure. In this paper, we have used the Pearson's correlation coefficient. However, other measures with the similar properties could be used for further study.

## Methods

### Datasets

In our experiments, we extracted the expression profiles of miRNAs and mRNAs from the experimental data previously published by Lu *et al. *[9]. This dataset consists of 217 miRNAs and about 16,063 mRNAs on 89 multiple human cancer samples. The current miRNA target prediction methods are mainly based on the principle of miRNA-target interactions, and the accuracy of these methods has been confirmed by experimental validation of randomly selected miRNA targets [33] and by large-scale gene expression profiling studies [34]. Though there are several available miRNA target prediction methods such as PicTar, miRanda, and TargetScan, a recent study indicated that PicTar had the highest success rate in target gene prediction [35]. Moreover, up to 90% of the randomly selected miRNA targets from the predictions by PicTar have been validated as true targets [33]. We thus utilized PicTar algorithm [12] for obtaining predicted target genes of each miRNAs.

From three kinds of data (expression profiles of miRNAs and mRNAs, and miRNA target genes), we analyzed the relationships among 121 human miRNAs and 801 mRNAs, which are linked together. Of these 801 mRNA × 121 miRNA possible binding pairs, 4,629 pairs with significant binding scores (PicTar's score ≥ 1.0) were used in our experiments. Specifically, one miRNA binds to 38.25 mRNAs and one mRNA is bound by 5.77 miRNA on average in our data set. Further information about the original datasets is shown in Table 5.

**Table 5:** Overview of the original datasets used in this paper

Dataset	Content	Amount	Reference
1	miRNA-target binding information	230 miRNAs 2410 mRNAs	Krek *et al. *[12]
2	microRNA expression profiles	217 miRNAs 89 samples	Lu *et al. *[9]
3	messenger RNA (mRNA) expression profiles 89 samples	16063 mRNAs	Lu *et al. *[9]

From the original datasets we analyze a set of 121 miRNAs and 801 mRNAs; 121 miRNAs are overlapping of miRNAs in the dataset 1 and the dataset 2; 801 mRNAs are overlapping of mRNAs in the dataset 1 and the dataset 3, the binding score (i.e. PicTar's score) of all interactions between miRNAs and mRNAs are not less than 1.0.

### Method overview

**Figure 2:**
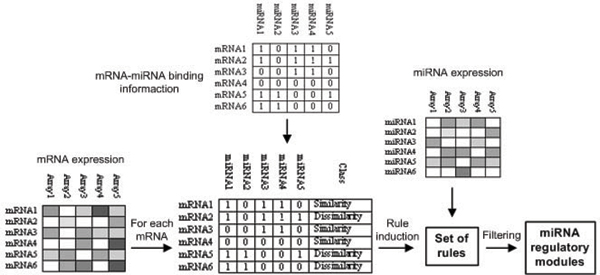
**Schematic description of our method for finding MRMs**. An overview of our rule-based method for finding miRNA regulatory rules from multiple information sources, including miRNA expression profiles, mRNA expression profiles, and miRNA-target binding information.

The problem can be formulated as follows: given a set of miRNAs (*mi*_1_, *mi*_2_,..., *mi*_*M*_) and a set of their target genes (mRNAs) (*m*_1_, *m*_2_,..., *m*_*N*_), we need to find a set of MRMs, each MRM is defined as a subset of miRNAs (*mi*_*i*1_, *mi*_*i*2_,..., *mi*_*ik*_) and a subset of target genes (*m*_*j*1_, *m*_*j*2_,..., *m*_*jl*_), where |*ik*| ≤ |*M*| and |*jl*| ≤ |*N*|. Figure 2 shows procedural steps of our approach. In the first step, we consider the first line (i.e. first gene) of the target gene (mRNA) expression profile table. We calculate the correlation coefficients between it and all other genes. The gene set will be divided into two classes, *similarity *and *dissimilarity *by using a correlation threshold. Next, we construct a regulatory decision table for the current gene by adding a class-column into the miRNA binding information table (Figure 2). We then apply the CN2-SD rule induction system [36] to produce a set of miRNA-mRNA regulatory rules. After that we use several filtering procedures to remove uninteresting rules. Only significant rules, which contain the miRNAs with highly correlated expression profiles, are considered to generate potential MRMs. This procedure will be repeated for the second gene in the mRNA expression profile table, and for all other genes.

### The Pearson's correlation coefficient

In statistics, the Pearson's correlation coefficient (PCC) is a measure of similarity/dissimilarity between two random variables. In our case, we use the PCC for measuring similarity/dissimilarity between expression patterns of two genes or two miRNAs. Given two genes *x *and *y*, the PCC of *x *and *y *is defined as follows:

(1) $$PCC(x,y)=\frac{\sum_{i=1}^{m}\left(x_{i}-\bar{x})(y_{i}-\bar{y}\right)}{\sqrt{\sum_{i=1}^{m}{\left(x_{i}-\bar{x}\right)}^{2}\sum_{i=1}^{m}{\left(y_{i}-\bar{y}\right)}^{2}}}$$

where *x*_*i *_and *y*_*i *_are the *i*th sample values of genes *x *and *y*, respectively; $\bar{x}$ and $\bar{y}$ are mean values obtained from *m *samples of genes *x *and *y*, respectively. The PCC of a pair of genes commonly returns a real value in [+1, -1]. *PCC*(*x*, *y*) > 0 represents that *x *and *y *are positively correlated with the degree of correlation. On the other hand, *PCC*(*x*, *y*) < 0 represents that *x *and *y *are negatively correlated with a value |*PCC*(*x*, *y*)|. A positive value of the PCC indicates that two genes are co-expressed and a negative value of the PCC indicates that opposite expression pattern exists between them. We can see that with this measure, genes with low- and high-expression values may be placed in the same cluster if they have similar patterns of changes in expression values over the samples. The advantage of the PCC over the Euclidean measure is that the Euclidean methods find mainly spherical shape of clusters, even if the shape of clusters may not be present in the dataset. The PCC is used as a measure of similarity/dissimilarity of cluster genes with similar expression patterns.

### Rule induction

Rule induction is a machine learning technique that has been successfully applied in subgroup discovery. The problem of subgroup discovery can be defined as follows: given a population of individuals and a property of those individuals we are interested in, find population subgroups that are interesting with respect to the property of interest [36]. The induced rules usually have the form *Cond → Class*, where *Class *is a value of the property of interest, and *Cond *is a conjunction of attribute-value pairs selected from the features describing the training instances. In our case, *Class *has two values, *similarity *and *dissimilarity*. Attributes are miRNAs and attribute-value is 0 or 1.

In general, there are three strategies for inducing rules (describing individual interesting patterns) from data: separate-and-conquer, divide-and-conquer and exhaustive search [37]. The separate-and-conquer strategy searches for a rule that covers part of its training instances, separates (or reassigns with lower weight) these examples, and recursively conquers the remaining examples by learning more rules until no examples remain. The divide-and-conquer strategy is used in decision tree algorithms; this strategy is restricted to learning non-overlapping rules only. The exhaustive search strategy explores almost all of the whole search space. The basic idea is to use an association rule algorithm to gather all rules that predict the class attribute and also pass a minimum quality criterion.

By implementation, the divide-and-conquer strategy (in decision tree-based algorithms) is restricted to learn non-overlapping rules only. The exhaustive strategy (in association rule-based algorithms) has the problem of producing many redundant rules. The separate-and-conquer algorithms can partially avoid these disadvantages [36,38], which is one of the main reasons for its popularity.

CN2 is a rule induction system implementing the separate-and-conquer strategy [39]. It learns a rule set by iteratively adding rules one at a time. Examples covered by the rule are removed from the search space before learning the next rule to add to the rule set. This is repeated until all examples are covered by at least one rule in the rule set or some stopping criteria is satisfied. Finally, CN2 can induce a set of independent rules, where each rule describes a specific subgroup of instances. This is not suitable for description tasks (discovering individual rules describing interesting patterns, as in this work). Since CN2 only induces the first few rules discovered are usually interesting. Subsequently induced rules are obtained from biased example subsets, i.e., subsets including only positive examples that are not covered by previously induced rules.

In 2004, CN2-SD, an improvement of CN2 for subgroup discovery, was proposed [36]. The CN2-SD generalizes the covering algorithm by introducing example weights. Initially, all examples have a weight of 1.0. However, the weights of examples covered by a rule will not be set to 0 (they are not removed as in CN2), but instead will be reduced by a certain factor. The resulting number of rules is typically higher than with CN2, since most examples will be covered by more than one rule. CN2-SD is, therefore, better able to learn local patterns, since the influence of previously covered patterns is reduced, but not completely ignored. In order to evaluate the rules with higher generality, CN2-SD also uses a weighted relative accuracy heuristic as presented in Equation 2. The weighted covering strategy tends to find rules that explain overlapped subgroups of instances in the search space, so the weighted relative accuracy heuristic produces highly general rules that express the knowledge contained in one specific subgroup. For these reasons, we utilize the CN2-SD in the rest of this paper for finding miRNA regulatory rules.

(2) $$h_{WRA}(Cond\to Class)=\frac{p(Cond)}{p(Class|Cond)-p(Class)}$$

### Filtering rules

Though the CN2-SD rule induction system uses a weighted covering strategy to restrict the redundancy of learned rules and guarantee the scanning of the whole search space, uninteresting rules are still produced [36,37]. Let us assume that our rule *r *has a form: IF [*Cond*] THEN [*ClassDistribution*]. Where *Cond *= [*miR*_1 _= *val*_1_∧*miR*_2 _= *val*_2_∧*miR*_3 _= *val*_3_∧...∧*miR*_*k *_= *val*_*k*_] and *Classdistribution *= [*p*, *n*] is the class distribution of examples covered by *r *(*miR*_*i *_is a miRNA and *val*_*i *_= 0 or 1). We have used several heuristics to filter out unexpected rules. First, we remove trivial rules, *r *is called a trivial rule if the number of positive examples covered by *r *is less than 2. The reason is that the miRNAs in this rule only coregulate one gene, it is a trivial case. Second, if there is any miRNA in the *Cond *part of a rule which has a value equal to 0, this miRNA does not bind to the target genes of the corresponding rule. We also remove such rules. Third, we calculate the correlation coefficient between all miRNA pairs which appear in the same module. If the correlation coefficient of any miRNA pair is less than a given threshold, that rule will also be removed. This heuristic allows us to find MRMs which are not only highly correlated on target genes, but also highly correlated on miRNAs with respect to expression profiles.

## Competing interests

The authors declare that they have no competing interests.

## Authors' contributions

DHT and KS defined the research question, designed and performed the experiments. DHT and TBH drafted the manuscript. All authors contributed to and approved the final version of the manuscript.
